# Decentralised Global Service Discovery for the Internet of Things

**DOI:** 10.3390/s24072196

**Published:** 2024-03-29

**Authors:** Ryan Kurte, Zoran Salcic, Kevin I-Kai Wang

**Affiliations:** Department of Electrical and Computer Systems Engineering, University of Auckland, 20 Symonds Street, Auckland 1010, New Zealand; z.salcic@auckland.ac.nz (Z.S.); kevin.wang@auckland.ac.nz (K.I.-K.W.)

**Keywords:** internet of things, service discovery, distributed systems

## Abstract

The Internet of Things (IoT) consists of millions of devices deployed over hundreds of thousands of different networks, providing an ever-expanding resource to improve our understanding of and interactions with the physical world. Global service discovery is key to realizing the opportunities of the IoT, spanning disparate networks and technologies to enable the sharing, discovery, and utilisation of services and data outside of the context in which they are deployed. In this paper, we present Decentralised Service Registries (DSRs), a novel trustworthy decentralised approach to global IoT service discovery and interaction, building on DSF-IoT to allow users to simply create and share public and private service registries, to register and query for relevant services, and to access both current and historical data published by the services they discover. In DSR, services are registered and discovered using signed objects that are cryptographically associated with the registry service, linked into a signature chain, and stored and queried for using a novel verifiable DHT overlay. In contrast to existing centralised and decentralised approaches, DSRs decouple registries from supporting infrastructure, provide privacy and multi-tenancy, and support the verification of registry entries and history, service information, and published data to mitigate risks of service impersonation or the alteration of data. This decentralised approach is demonstrated through the creation and use of a DSR to register and search for real-world IoT devices and their data as well as qualified using a scalable cluster-based testbench for the high-fidelity emulation of peer-to-peer applications. DSRs are evaluated against existing approaches, demonstrating the novelty and utility of DSR to address key IoT challenges and enable the sharing, discovery, and use of IoT services.

## 1. Introduction

As the Internet of Things (IoT) continues to grow, from small-scale household deployments to larger projects that span organisations, cities, or countries, these systems provide ever-increasing opportunities to improve our understanding of and interactions with the physical world. However, in the existing IoT, devices and systems are isolated and siloed within specific organisations and ecosystems without any mechanisms for sharing, discovering, or interacting with services outside this context [1]. We believe global service discovery is key to realising the opportunities of the IoT by providing end-to-end approaches that enable the safe and reliable discovery and use of contextually relevant services to compose new applications and achieve their goals [2]. For example, in the modern world, many organisations and individuals deploy environmental monitoring services using a variety of protocols and networking technologies connected to internet infrastructures for brokering and data storage. In the future, global registries should allow users to discover relevant services across the IoT, such as air quality sensors deployed by different organisations across a city or country, or temperature and humidity sensors within a campus, and to compose and interact with these services, accessing current and historical sensor data or (if authorised) affecting the state of actuators.

However, global service discovery comes with several challenges. The IoT is heterogeneous, with devices deployed in different physical locations using a wide variety of networks, protocols, and supporting infrastructure [3]. Following the previous example, a personal weather station might be connected directly to the cloud-based infrastructure via a home Wi-Fi network while air quality sensors deployed across a city may be connected via low-bandwidth mesh networks bridged to the internet using gateways. Global service registries must support discovery and interaction, addressing key IoT challenges including service identification, specification, and communication to allow users to access data from and assert control over discovered services on disparate networks. Registries must also be scalable to support ever-growing numbers of IoT devices and users, and they must be reliable, providing fault tolerance and supporting increasingly critical applications [4]. To enable future IoT applications, global registries must support trust and privacy, providing an end-to-end verification of registry operation, resolved services, and service data, as well as allowing public or private use [5,6].

In the existing IoT, service discovery is primarily supported using centralised service registries that are deployed at a home or organisational level. These registries must be deployed as a part of the supporting infrastructure for an IoT system, alongside brokers for communication between services and databases for data storage and access, posing a substantial technical barrier to entry as well as requiring ongoing maintenance and support. Centralised registries support complex queries to allow services to be discovered within the registry; however, they are typically isolated within their individual or organisational context, without mechanisms for the sharing or coordination of registries or interaction with services and access to data. Decentralised registries offer a valuable alternative to centralised approaches, providing users and device vendors with an alternative to the cost and complexity of maintaining centralised registry infrastructure. Decentralised approaches offer improved scalability and reliability by distributing processing and storage requirements over networks of communicating peers. However, these approaches also introduce new challenges. Where the performance of centralised registries is predominantly a function of the underlying infrastructure, the operation of a decentralised registry depends on the coordination of and network communication between large numbers of peers, increasing the challenge of qualifying the correct operation and performance of decentralised approaches. Due to the use of public and untrusted peer-to-peer (p2p) infrastructure, it is also critical to provide mechanisms for trust and privacy, ensuring the integrity and confidentiality of data transiting the peer-to-peer network and allowing for discovered services and data to be used for real-world applications [7].

In this paper, we present Decentralised Service Registries (DSRs), a novel decentralised end-to-end approach to global service discovery and interaction simplifying the creation and sharing of trustworthy service registries to enable new applications using the IoT. Our approach builds on the service specification and interoperability provided by the Distributed Service Framework (DSF) [8] and DSF-IoT [9] to allow users to dynamically share, discover, and interact with contextually relevant IoT services using DSFs common distributed infrastructure. DSRs utilise a novel S\Kademlia-based [10,11] Distributed Hash Table (DHT) overlay supporting multiple registries and private or zero-knowledge queries while retaining the verifiability of overlay objects. In contrast with existing works, DSRs provide an end-to-end mechanism for discovering and interacting with services while addressing the need for trust and privacy. The adoption of cryptographically derived service and registry identifiers enables verifiable linking between registries, services, and data, mitigating the risks of service impersonation or data alteration. Alongside this, DSRs’ use of signature chains provide registry integrity and non-repudiation as well as support historic queries and the evaluation of registry operation to establish trust. DSRs’ use of decentralised infrastructure provides a scalable and reliable alternative to centralised approaches. This allows users to simply create and share registries, execute context-based queries to locate relevant services based on well-defined properties such as physical location or endpoint types, and interact with these services to subscribe or query for current and historical data using DSFs peer-to-peer network.

This paper consists of five key contributions:

- First, we identify key IoT challenges relating to global service discovery, highlighting the advantages and limitations of existing centralised and decentralised service registries.
- Second, we introduce DSRs, detailing the novel DHT and signature chain-based approach to the provision of trustworthy public and private decentralised service registries.
- Third, we demonstrate the use of DSR to discover real-world IoT devices. This illustrates the end-to-end operation of DSRs, simplifying the creation and sharing of registries that allow users to discover and interact with contextually relevant services.
- Fourth, we develop a novel cluster-based testbench for the emulation of peer-to-peer applications using commodity hardware. This testbench is used to qualify the operation and performance of DSRs with varying peers and network conditions.
- Finally, DSRs are evaluated against existing approaches to demonstrate the suitability of decentralised registries to address key IoT challenges and meet the need for global service discovery in the IoT.

## 2. Background and Related Works

The context-based global service discovery is key to enabling the real-world sharing of IoT services and data by allowing users to share, discover, and interact with services that are relevant to them. Global discovery mechanisms are those spanning physical and network environments that allow for the sharing of services outside of the physical, logical, and organisational siloes in which they are deployed. These discovery mechanisms must support contextual queries, expanding on network-based discovery mechanisms to include metadata such as service and endpoint types or real-world locations to allow users to search for relevant services to meet their needs. Registries must also address the need for access to discovered services and data, allowing users to compose services and retrieve current and historical information to achieve their goals. To support these end-to-end interactions, approaches to global service discovery must address the following key IoT challenges:

- Identification, providing globally unique and verifiable addresses allowing for a service to be consistently identified independently of physical location or underlying network technologies [12,13,14].
- Specification, providing unambiguous context-rich descriptions of services and data [15,16].
- Communication, enabling interaction with services across disparate physical and virtual environments including access to current and historic data [16,17,18,19,20,21].
- Scalability, suitable for use with ever-growing numbers of IoT devices and services [12,17,18,19,22].
- Reliability, supporting the operation of IoT devices in increasingly critical contexts [18,19,22].
- Trust and Privacy, allowing for operation and data to be verified while ensuring these are accessible only by authorized parties [12,14,18,19,21,22].

**Figure 1:**
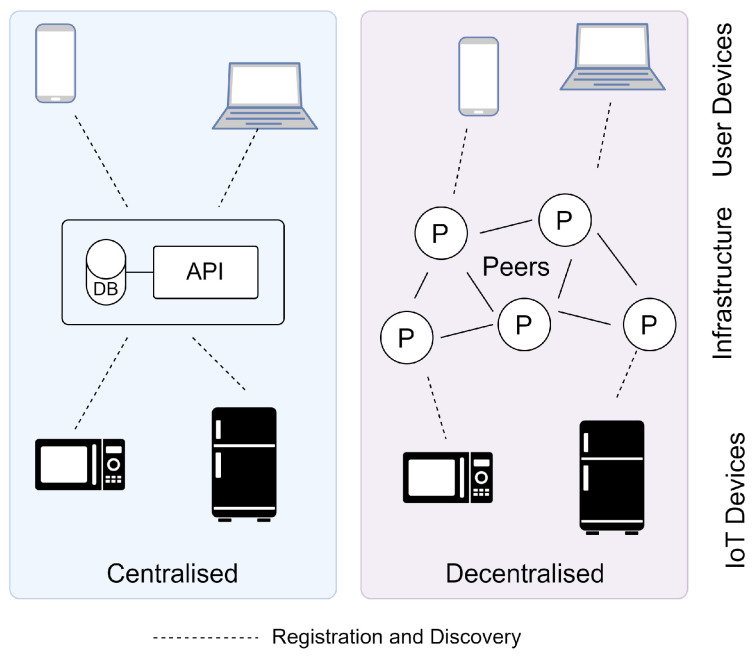
Centralised and decentralised registry infrastructure.

Service registries can be split into two key categories. Centralised approaches whereby registries consist of a database and an API server deployed by a vendor or organisation with a single point of contact for registration and discovery. Furthermore, decentralised approaches where registries utilise databases deployed across peer-to-peer networks, distributing storage and computation across peers while providing each peer with access to the registry. Figure 1 illustrates the communication between user and IoT devices with centralised and decentralised registries, highlighting the network of peers (P) supporting the latter case. Note that for decentralised approaches, both the user and IoT devices must typically either operate as peers or utilise existing peers to interact with the registry.

### 2.1. Centralised Approaches

In the existing IoT, high-level discovery is typically achieved using centralised registry servers. These provide APIs for service registration and discovery, a database for storing registered services, and mechanisms for querying using the service database. The IETF framework utilises the Constrained RESTful Environments (CoRE) Resource Directory [23]. Services dynamically discover resource directories using DNS Service Discovery (DNS-SD) [24] and then register their endpoints in the directory to allow these to be discovered by other users. CoRE resource directories support attribute-based queries; however, Constrained Application Protocol (CoAP)-based [25] approaches do not provide unambiguous service specifications or include mechanisms to describe service context [26]. The W3C defines the WoT Discovery process [27], with an optional Thing Directory to support service registration which enables querying for services. This approach is split into two stages: an introduction phase where devices discover relevant directories, and an exploration phase where Thing Description (TD) [28] is fetched and processed by the registry. To register a service, a device discovers available local directories and then prompts registration, allowing for the directory to fetch information for the service and store this for future querying. Searching is supported using syntactic or SPARQL-based [29] queries over registered services. Jia et al. [30] present an ontology-based approach to semantic service matching in the IoT, with a focus on improving query performance. QoDisco [31] supports semantic service discovery based on the Semantic Sensor Network Ontology (SSNO) [32], utilising coordinating repositories to store resource models and device data unified by a common API and supporting queries via SPARQL. QoDisco supports combining multiple repositories to improve scalability and fault tolerance, allowing for registrations to be split between servers; however, it fails to address the challenges of identity or interoperability as required for global use.

These centralised registries are deployed in isolation and are operated at a personal or organisational level without mechanisms for interoperation between registry hosts or higher-level coordination across registries. Kamilaris et al. [2] highlight the importance of registry interoperability, proposing the use of linked data to create a higher-level search engine for IoT services using web-crawling, isolated but internet-connected registry instances. For global use, registries must address this need for coordination by providing common interfaces for coordination between registry instances. Deploying centralised registries also requires the integration of external security mechanisms to provide authentication and authorisation. Global registries should also support multi-tenancy and privacy, decreasing the complexity of creating registries by allowing for multiple registries to utilise shared infrastructure while ensuring only authorised users can interact with a given registry. For users to effectively utilise discovered services, these centralised registries must be paired with infrastructure for communication with services as well as the storage and analysis of data, which must be deployed and maintained by device vendors.

### 2.2. Decentralised Approaches

Decentralised registries offer a valuable alternative to more common centralised approaches, replacing centrally controlled infrastructure with communicating networks of peers. Decentralised registries typically utilise a distributed database such as a Kademlia [10] Distributed Hash Table (DHT) to store and resolve service information paired with a specification for the description of IoT services. DHTs assign unique hash-based identifiers (IDs) to peers and data and use the logical distance between these identifiers to partition data storage and routing information, providing a key–value interface for storing and retrieving values. To interact with the DHT, a peer first performs a recursive search to locate peers nearest to the identifier of interest, followed by a request to store or fetch data using these nearby peers. This peer-to-peer approach exacerbates the challenges of trust and privacy, requiring mechanisms for authorisation to control who can store data at a given identifier, as well as confidentiality so that private data cannot be accessed by unauthorised peers. Secure Kademlia (S\Kademlia) [11] modifies Kademlia through the use of cryptographically-derived peer identities by using signatures to ensure peers can only publish objects at their own address. DHTs are often extended through the use of overlays, where hashing is used to translate between values and IDs, allowing for the construction of searchable indexes using the underlying key–value store.

These overlays enable the use of DHTs for IoT service discovery, supporting the creation of searchable [33] and contextual or location-aware service registries [34]. However, as overlays involve storing objects at arbitrary IDs, these are typically incompatible with S\Kademlia-like approaches to identity and verification. Cirani et al. [35] demonstrate an approach to the global discovery of CoAP-based services through the use of proxying gateways that provide a peer-to-peer DHT for service registration and discovery. To communicate with a service, a user first queries the overlay to find the appropriate gateway address; then, the user issues requests to the service via the proxying gateway. The use of CoAP with CoRE provides an efficient service encoding and enables endpoint discovery while proxying gateways allow for access to services across different networks. However, this approach fails to address the need for trust and privacy of both services and registries, while the lack of a complete and unambiguous specification provides limited interoperability with services and elides mechanisms for trust or access to historic service data. Zhang et al. [36] propose the Global Data Plane (GDP), a Merkle-Tree-based append-only database as an intermediate format for IoT data. This approach uses content-based service addressing and supports the storage and querying of historic data. However, it fails to address the need for service discovery. Kamel et al. [37,38] construct a DHT-based overlay to enable attribute-based service discovery using a Region-based DHT (RBDHT). This includes mechanisms for private registration and masking queries by eliding the lower bits of DHT identifiers during DHT operations, as well as the enforcement of access policies using Attribute Based Encryption (ABE). However, it does not address trust or multi-tenancy as well as requiring the deployment of attribute CAs to manage credentials. Zorgati et al. [39] present a two-layer approach to decentralised service discovery by first clustering objects using a Semantic Overlay Network (SON) and then by federating gateways using a DHT. This approach improves discovery performance by allowing for queries to be routed to relevant clusters; however, it fails to support communication or address the need for trust and privacy. Tanganelli et al. [40] present DHT-based discovery using CoAP and CBOR-based gateways, highlighting the importance of pairing discovery and *access* mechanisms to allow for users to interact with the services they discover. Gateways provide access to registered services; however, again, this approach fails to address the need for access to historical data, trust, or privacy. Tang et al. [41] propose a discovery mechanism using the IoTA blockchain, highlighting the utility of a secure and immutable ledger for service registration. However, while IoTA’s tangle improves on the throughput limitations of classical blockchains, the consensus algorithms used in blockchain-based technologies offer low performance while demanding substantial bandwidth and processing capacity, rendering these broadly unsuitable for IoT applications.

### 2.3. DSF and DSF-IoT

The Distributed Service Framework (DSF) [8] is a platform for building and deploying decentralised applications. DSF utilises an S\Kademlia-based DHT for storing and resolving service information alongside a peer-to-peer mechanism for publishing, subscribing, and querying for service data. The DSF network consists of many communicating peers executing a standard daemon application to provide a common underlying infrastructure for the deployment of decentralised services. The daemon provides an Application Programming Interface for operations using the distributed network, while client libraries and command-line tooling build on this API to simplify the use of DSF and the development of DSF applications. Users and other applications interact via the daemon to utilise the network for service resolution and interaction, providing a peer-to-peer alternative to the message brokers and databases that are typically used in centralised IoT infrastructure.

DSF services utilise unique Service IDs (SIDs) derived from a cryptographic public key, providing content-based network- and location-independent addresses. These provide a verifiable and globally unique alternative to commonly used URLs and IP-based identifiers to support mobility and cross-network service interaction [42]. Services in DSF publish service information using pages that are stored in the DHT to support service resolution and replication, as well as data using data blocks that are distributed between subscribing peers to support publishing and subscription. This allows for users to resolve SIDs to service information and then to access and interact with services using a peer-to-peer infrastructure for communication and storage. Pages and blocks are signed by the publishing service and linked to the previous object to create an immutable signature chain [43] of objects associated with a service to support verification and non-repudiation. DSF supports privacy through the use of partial encryption whereby sensitive fields are encrypted prior to object signing, allowing for objects to be distributed and verified while limiting access to privileged information to authorised parties. The signing and encryption of objects allows subscribers to store and re-publish service data enabling the safe replication (or mirroring) of services across the peer-to-peer network, providing scalable and reliable access to service data while maintaining end-to-end privacy and verifiability of published objects.

DSF-IoT [9] extends DSF with an efficient and unambiguous specification for the description of IoT services, providing a basis for the development and deployment of peer-to-peer IoT devices and applications. IoT services are represented as a set of endpoints with associated kinds and units (for example, temperature in °C) coupled with metadata that describe contextual information for local and global discovery use. Services publish Service Page (SP) containing endpoint and context information that is stored and retrieved using the DHT, alongside blocks containing endpoint data that are stored and distributed between subscribing peers. DSF-IoT provides a consistent location- and network-independent mechanism for interacting with IoT services, from address resolution using the DHT to subscribing to real-time data or querying for historic data using the peer-to-peer network. However, prior to the introduction of DSRs, this fails to address the need for global service discovery, requiring users to distribute SIDs or use network-based approaches to discover local services.

### 2.4. Research Opportunities

While there is a variety of research on global service discovery using both centralised and decentralised approaches, existing works fail to address the need for trustworthy, private, end-to-end mechanisms for discovery and interoperability. Future approaches must support multi-tenancy to simplify the creation and use of registries by decoupling these from supporting infrastructure as well as privacy to enable authentication and the use of public and private registries. These approaches should simplify the creation and sharing of registries while meeting the need for scalability and reliability to suit the growing IoT. New approaches to global discovery must also provide access to discovered services, allowing users to execute historical queries as well as to fetch both current and historical service data. For example, to fetch climate data for an area over the last year, it must be possible to search for both currently and historically registered services and then to fetch historical data associated with currently and previously active discovered services. New registries must also provide trust, ensuring services can only be registered by authorised parties, allowing registry operation to be validated, and providing provenance for discovered services and data.

## 3. Decentralised Service Registries (DSRs)

In order to address the need for global IoT service discovery, we introduce Decentralised Service Registries (DSRs), extending DSF and DSF-IoT to support the creation and sharing of global service registries using a common decentralised infrastructure. DSRs provide a trustworthy end-to-end approach allowing for IoT services to be safely registered, discovered, and interacted with by authorised parties using context-based queries without the cost or complexity of deploying a centralised registry infrastructure. The use of DSFs globally unique and verifiable service identities allow for IoT services to be consistently identified regardless of physical location or underlying network technologies, while the underlying peer-to-peer infrastructure enables mobility and cross-network communication, allowing users and services to interact using these identities. The DSF-IoTs specification provides an unambiguous and context-rich mechanism for describing IoT services that enable context-based querying as well as human and machine interpretation of service information and data. Altogether, this provides an end-to-end approach to global IoT service discovery, enabling the creation and sharing of registries which permit users to search for contextually relevant services, to resolve these to meaningful service information, and to interact with the services they discover.

DSRs are identified by Service ID (SID) and are separated using namespaces to support the use of multiple and overlapping registries. In contrast to hierarchical approaches to service resolution (such as DNS), this namespacing allows individuals to compose a set of authoritative sources by choosing a set of registries they trust. An organisation may elect to provide an authoritative registry, for example, “organisation.com” to match their web presence, while individuals are free to create overlapping “home” or “local” registries and to select from available registries to meet their needs. To interact with a registry, users must have an associated peer which executes the DSF daemon and provides an APIs for discovering and managing services. This peer communicates with other peers to maintain the S\Kademlia-based DHT as well as to distribute services and data. Where centralised registries require registration and searches to be issued using a single point creating a single point-of-failure and storage and processing bottlenecks, our decentralised approach allows any peer with appropriate knowledge of the DSR to execute queries and resolve services using information distributed across the network. This peer-to-peer approach provides scalability and resilience, distributing the communication and storage requirements across available peers while only requiring the DSR for maintenance and periodic re-issuance of the index. Where existing registries must be paired with external mechanisms for security and privacy, DSRs’ use of signed objects and signature chains supports the evaluation of registry trust, while encryption and derivation mechanisms ensure privacy, enabling the safe co-existence of multiple public and private registries using shared peer-to-peer infrastructure. Registries can be simply created and adopted on any peer via the daemon APIs or command-line tooling, while it is expected that future applications will provide user-friendly interfaces using these APIs.

To enable this registry operation, the DSF distributed database has been extended with support for a novel searchable overlay, using a linked approach to enable field-based indexing and querying while maintaining the end-to-end privacy and verifiability of services and objects in the database. To create these overlays, we introduce a new tertiary page type for storage in the DHT. Where existing DSF primary pages providing service descriptions are stored at the address of the service they describe, and secondary pages supporting annotation (for example, service replica advertisements) are stored at the address of the service they target, these new tertiary pages are stored in the database at a deterministic Tertiary ID (TID) derived using the identity of the publishing DSR and the value of the field to be indexed. DSRs construct an overlay by storing these tertiary pages in the DHT to provide an index linking TIDs to services matching a given query.

TIDs are derived using a hash of the field to be queried (for example, “room = office”) combined with the identity of the DSR. This derivation provides a deterministic mechanism to convert queries into TIDs while ensuring TIDs that belong to different registries are normalised across identifiers (and, thus, peers) in the DHT. For public service registries, the registry public key ($SR_{pub\_key}$) is used as a seed for data hashing as per Equation (1). The one-way hashing of field values allows for queries to include private information without leaking this to users or registries as well as enabling zero-knowledge registration of services by providing a registry with pre-hashed field values. However, due to the deterministic derivation process, it may be possible to brute-force low-entropy private field values represented by a TID within a public registry. Including the registry public key in the derivation process increases the cost of these attacks by ensuring TIDs are unique for each public registry. Private registries utilise key derivation based on the existing registry secret key ($SR_{sec\_key}$, a credential providing access to private services) as a seed to support secure hashing, as per Equation (2). This use of secure hashing mitigates the risk of brute-force collision attacks as TID derivation requires access to the service secret key, while service links are encrypted and can only be resolved by users with access to these credentials. This approach provides a basic authentication mechanism to ensure only authorised parties can infer the location or contents of entries in a private registry, while allowing for private registry pages to be safely shared between peers using the DHT.

(1) $$TID=hash(SR_{pub\_key}+\;hash\left(field\right))$$

(2) $$TID=hash(derive\left(SR_{sec\_key}\right)+\;hash\left(field\right))$$

**Figure 2:**
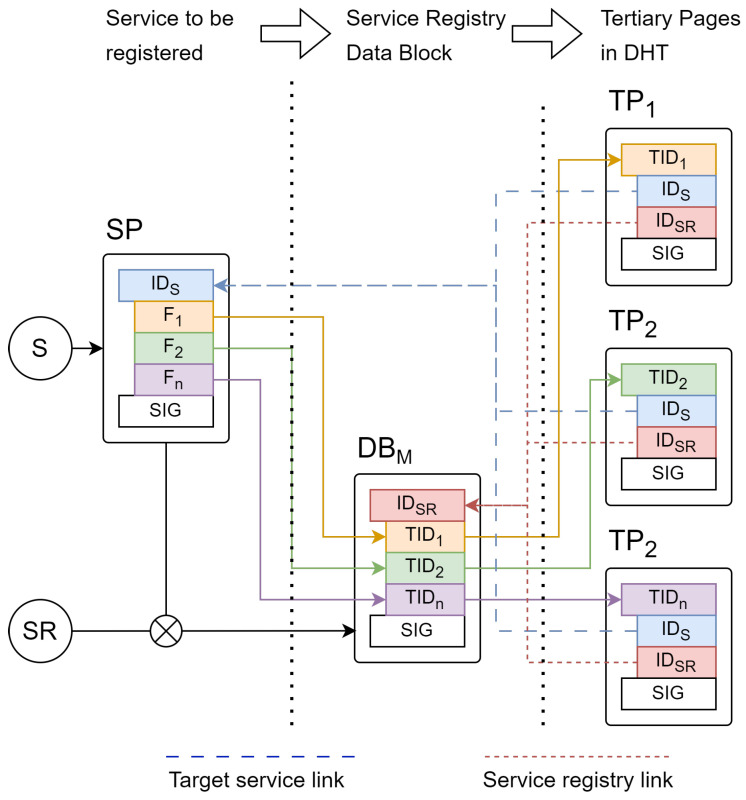
Service registry object relationships.

To register a service, the DSR is provided with a Service Page (SP) describing the service to be registered, including endpoint types and associated metadata. For example, an office environmental sensor may have endpoints for temperature, pressure, and humidity, as well as a friendly name for the device and a room location. The DSR then derives a TID for each of these fields and publishes a Data Block (DB) containing the target service ID and generated TIDs, creating a verifiable signature-chain of records provided by the registry. This signature chain provides an immutable history of registry operations similar to the Certificate Transparency (CT) logs issued by TLS Certificate Authorities (CAs), allowing for the operation of the registry to be validated as well as enabling queries using the historical registry state. Finally, the DSR generates a Tertiary Page (TP) for each TID containing the SIDs of the linked service and publishing DSR as well as a link to the associated DB, and it stores these in the database at the appropriate TID. Each TP contains an issuance and expiry date allowing for these to be validated and automatically removed from the DHT on expiry, and must be periodically re-published by the DSR to maintain the registry. To refresh or update registry entries, a DSR will publish a new DB and store the new TPs in the database, automatically replacing previous service information. Figure 2 illustrates this relationship between an SP containing *n* queryable fields ($F_{n}$) that describe a service (S) for registration, the DB published by the DSR that contains the derived TIDs for each field, and the *n* TPs generated and stored in the database for each TID by the DSR. Subscribers to the DSR can use any indexed field to search for matching services in a registry. This occurs, first, by generating a TIDs for the query by combining the field value and DSR information, and then looking up matching TPs using the distributed database, and, finally, using each discovered TPs to look up service and replica pages to resolve these to service descriptions and available replicas for further interaction.

Where centralised registries store all service information at a single point and require a single round-trip interaction with the registry service for registration or searching, interactions with DSRs consist of several high-level fetch or store operations to resolve or emplace data into the DHT, while service information is distributed across the network of peers. Each DHT operation consists of a recursive search for peers near the identifier of interest, followed by an operation to obtain or put data using a subset of the closest peers to the target identifier. DHTs operations utilise a configurable number of peers for each operation, ensuring data are stored at multiple locations to provide redundancy at the cost of an increase in storage and maintenance bandwidth. The S\Kademlia-based DHT used by DSF has two key parameters for tuning the operation of the algorithm:

- *k*—the size of *KBuckets* (and, thus, the size of the routing table) used to store peer routing information;
- α—also known as concurrency, or the number of parallel nodes used for each operation.

Increased values of *k* will increase the size of the routing table and the cost of maintaining routing information while decreasing the number of iterations required to perform a search for a given network size. Increasing α decreases the number of iterations at the cost of increased network traffic (and latency) and improves resilience as a given data value will be stored across more peers in the DHT.

DSRs allow any peer with the appropriate credentials to perform a search operation using any registry while distributing storage and query operations over available peers for scalability and reliability. However, decentralised registry operations require internet connectivity for peer-to-peer communication, even where the querying peer and matching devices exist on the same local network. Due to the partial nature of the routing information available to a DHT, the number of recursive iterations required to complete an operation depends on factors such as the routing algorithm, configuration, and size and performance of the network. To efficiently support the registry operation, the S\Kademlia-based DHT is modified to allow for multiple values to be stored at a given identifier. For example, both service and replica information objects are located at the SID of the target service, while a DSR will store multiple tertiary pages for a given query at the same TID. This is in contrast to typical DHT overlays where annotations are offset to ensure a 1:1 relationship between keys and values, thus requiring an unbounded number of lookups for each possible offset, improving the efficiency of registry operations at the cost of some reduction in resilience, as related data for a TID will be stored on the same subset of peers. Registering a service, thus, requires one DHT store operation for each corresponding TID, while searching requires one fetch operation to discover *N* matching services at a TID followed by further *N* fetch operations to resolve information for each matching link.

Tertiary pages are signed by the publishing DSR and linked to the relevant DB, allowing for these to be verified both on insertion into the DHT and when resolved during querying. As described earlier, these pages may also be encrypted to ensure only authorised parties can resolve links to registered services. As Tertiary Pages must be created and stored for each searchable field in a service, it is likely that the number of TPs will substantially exceed those of primary or secondary pages. To minimise storage impact and opportunities for abuse, TPs may not contain any application-defined data and, like SP, must be periodically re-issued by the publishing registry to avoid expiry. While the DHT naturally provides redundancy to mitigate churn (the disconnection and reconnection of peers), DSRs also periodically check the reachability of registered services and issue updates to ensure TPs are stored in the DHT with the required level of redundancy.

## 4. Demonstration

In order to illustrate the end-to-end operation of decentralised service registries, we demonstrate the creation, sharing, and use of a DSR by a pair of users interacting with real-world IoT environmental sensors via DSFs command-line tooling. These sensors consist of a Raspberry Pi Zero 2W paired with a variety of different environmental sensors with endpoints including temperature, humidity, pressure, and CO^2^ concentration. Each sensor publishes a DSF-IoT service and is deployed in a different room in an office building. Where centralised approaches provide a single point of contact for interacting with a registry service, decentralised registries are distributed across the network of peers. Each user has an associated peer that executes the DSF daemon, communicating with existing DSF peers to maintain the DHT and distribute services and data as well as providing an API for the discovery and management of services. For the purpose of this demonstration, each user interacts with their peer via command-line tools built on these APIs.

**Figure 3:**
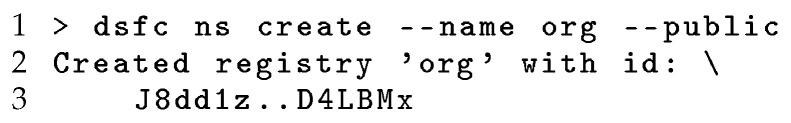
Creating a DSR.

**Figure 4:**
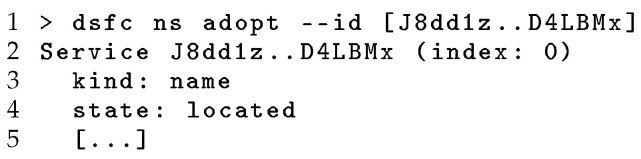
Adopting a DSR.

**Figure 5:**
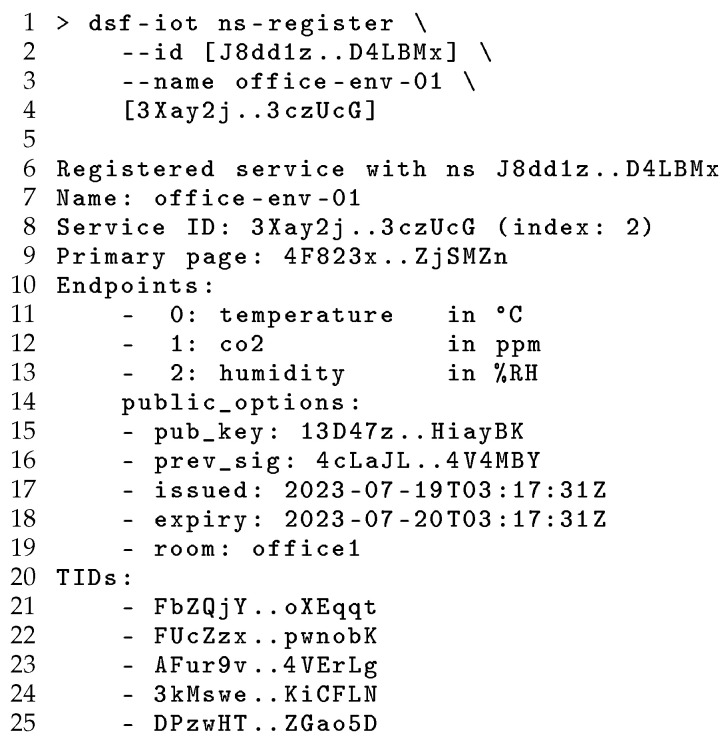
Registering a service using a DSRs.

To set up the registry, the publishing user creates a public DSR using the namespace “org” which is registered in the DHT as illustrated in Figure 3. Once the registry has been created, this can be adopted by other users via the registry ID, as shown in Figure 4. Note the simple creation and sharing of a DSR without the deployment or configuration of supporting infrastructure as is required for existing centralised approaches. Following the creation of the registry, the dsf-iot utility can be used to register each IoT service, generating TIDs for each endpoint kind and metadata field and then publishing the DB containing this information and storing the corresponding TPs in the DHT to construct the overlay. This registration process is demonstrated in Figure 5, highlighting the TIDs generated from the indexed fields and DSR information. As discussed in Section 3, each of these TIDs is unique to the combination of DSR and the value of the indexed field, while multiple TPs may be published to a single TID for each service with a matching field.

**Figure 6:**
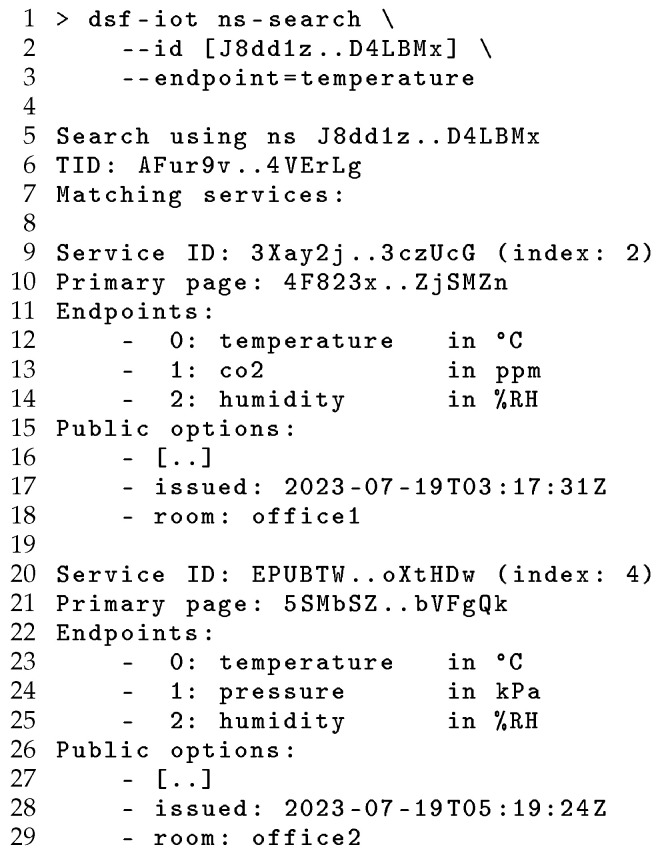
Searching for services using DSR.

To search for services, the discovering user must first be provided with the registry ID (and for private registries, the appropriate credentials) to locate and establish trust with the DSR. Figure 4 shows this adoption process, using the SID generated at registry creation to look up registry information using the DHT. The use of cryptographically derived service identifiers ensures unauthorised parties cannot impersonate a registry with a given SID. To search the registry, an DSR is selected by name or SID, and then a TID is generated for the query, and a search is performed for tertiary pages at this location. These tertiary pages are then resolved to service IDs, which can be used to query for Service Pages containing descriptions of the matching services and Replica Page describing available replicas for further communication. Figure 6 demonstrates this use of a DSR to search for registered services with temperature endpoints. This occurs, first, by deriving a TID for the query and then locating a pair of TPs matching this TID, and, finally, fetching and decoding the information for each discovered service to provide this to the user. Again, the SID-based linking between TPs and SP mitigates the risk of service impersonation.

**Figure 7:**
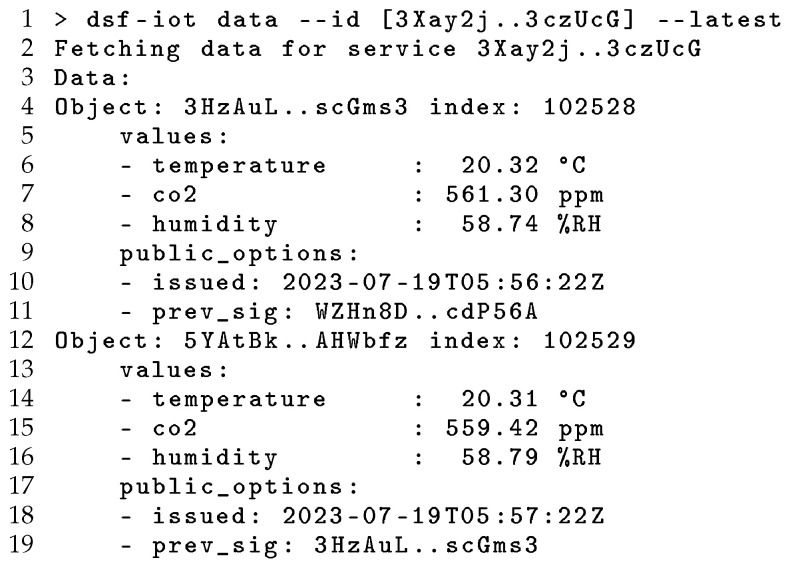
Fetching the latest data from a discovered IoT service.

Following this search operation, users can interact with discovered services using the resolved SIDs by subscribing or issuing requests for data, while DSF manages communication with the target service or available replicas utilising the distributed network. Figure 7 illustrates this operation using the CLI to request data from one of the services discovered in the previous example, which is decoded and displayed to the user. This provides an end-to-end approach to global service discovery and interaction, addressing the need for discovery mechanisms that integrate context-based querying with unambiguous specifications to describe services and data, and then using a DSF-IoT replication layer to access current and historic data published by discovered services. Published data objects are signed by the publishing service to provide integrity protection and linked into a signature chain to provide non-repudiation. In contrast with existing centralised and decentralised approaches, DSR provides trust and privacy, cryptographically associating registry entries with services and their data to allow users to verify the operation of registries and the results of their queries.

## 5. Performance and Operation

Verifying the correct operation and performance of peer-to-peer systems remains a substantial challenge, requiring the simulation or emulation of large networks of peers to replicate real-world deployments. Performance is also a key concern in decentralised systems due to increased communication and networking overheads when compared with centralised approaches. In order to evaluate the operation and performance of DSRs, we developed a scalable cluster-based testbench for the deployment of a virtual peer-to-peer infrastructure, decoupling peers and networking from physical hardware to allow for networks of varying sizes and configurations to be programmatically deployed and evaluated using commodity hardware. This testbench is used to demonstrate the correct operation and measure the real-world performance of search operations using DSRs with varying network sizes and latencies modelled to reflect best- and worst-case internet operations, illustrating the suitability of DSRs as an alternative to existing centralised and decentralised IoT service registries.

**Figure 8:**
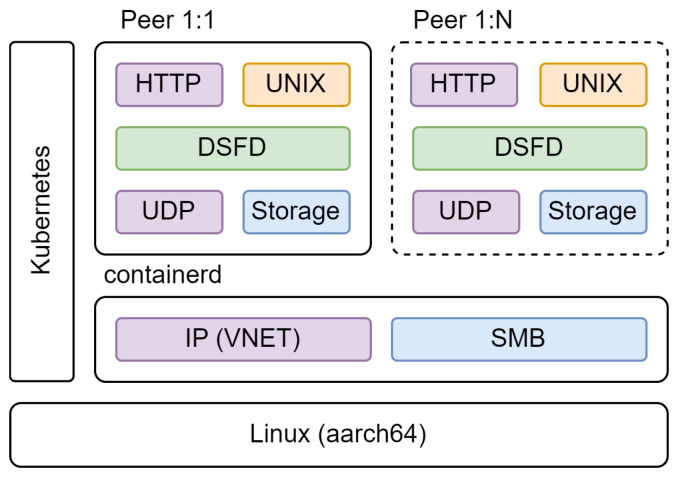
Testbench software architecture.

The testbench consists of a cluster of Raspberry Pi hosts communicating via an IP-based network. Raspberry Pis were selected as a reasonably available and standard host platform for clustering to provide an affordable, if less performant, alternative to ×64 computers, with the cluster consisting of 4× Raspberry Pi 4 and 4× Raspberry Pi 3 devices running 64-bit Debian Linux (6.1.21). Kubernetes was used for cluster orchestration to manage the deployment of application containers across available hosts as well as the resources available to each container. Containerd was utilised for isolation and virtualisation, providing a virtual IP network for communication between containers to emulate peer-to-peer connectivity via the internet as well as network-based storage to allow each peer to write and retrieve persistent information. Each peer executes a containerised version of the full DSF daemon which communicates with other peers using UDP packets and exposes an HTTP control API for use by the test harness. The Kubernetes API provides a programmatic mechanism to deploy and manage networks of peers deployed using the cluster. An overview of this software architecture is provided in Figure 8.

**Figure 9:**
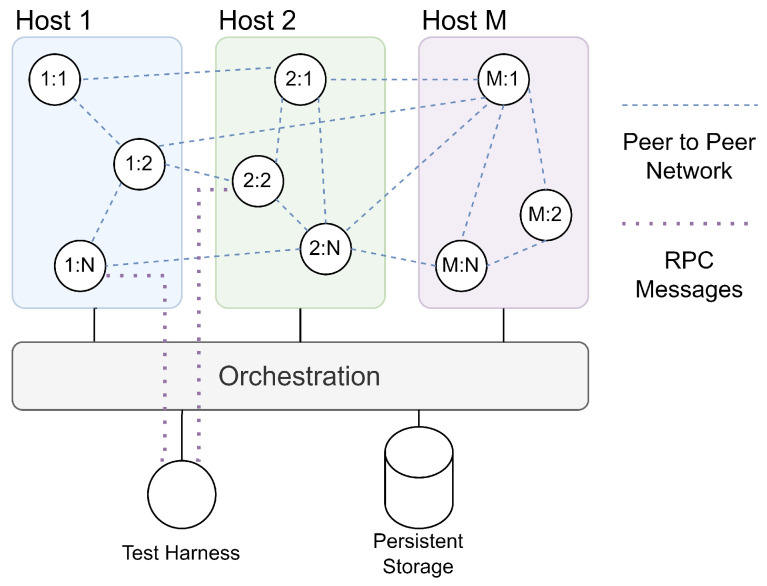
Testbench network.

Where decentralised systems are predominantly evaluated using abstract models or high-level simulation, offering high scalability with relatively low fidelity to support the simulation of large-scale networks [44,45], this approach provides a high-fidelity model of the distributed system to enable both performance and operational evaluation, including application, communication, and operating-system concerns at the cost of some scalability due to the increased cost of executing the full peer environment. The test environment consists of a network of peers deployed onto the cluster using the orchestrator, each interacting to support the operation of the distributed network. A test harness then interacts with simulated peers using the HTTP control API to configure and evaluate the operation of the distributed system. This network is illustrated in Figure 9, consisting of up to *M* hosts, each executing up to *N* peers managed by the orchestration layer, with the external test harness interacting with each peer. A hypothetical virtual network for peer-to-peer communication is highlighted with examples of the cluster networking paths used to support this, though it is important to note that routing is a function of the generated identities of each peer and the operation of the DHT.

As discussed in Section 3, the performance of a decentralised registry depends both on the size of the network of peers and communication latencies between each pair of peers. To evaluate this performance tests are executed for network sizes from 50 to 250 peers with injected network latencies to reflect peer-to-peer communication over the internet. For the purpose of this evaluation, injected latencies are constant between any two peers with 50 ms selected to represent a nominal average and 100 ms as a worst-case average for internet communication [38,45], and 0 ms provided as a best-case baseline. Real-world networks will likely experience a variety of communication delays between peers depending on physical location and network technologies; however, due to the stochastic nature of the DHT modelling, these point-to-point communication links in more detail would not significantly improve the fidelity of the simulation. Each test uses a single DSR with a constant set of services for searching. A test harness is used to emulate user interaction with the DSR, first by configuring an DSR and registering the set of services for discovery, and then by interacting with each peer to execute a search operation for each registered service. Each test consists of the following sequence:

1. The required number and configuration of peers are deployed onto the cluster as a Kubernetes service.
2. The network is bootstrapped by instructing each peer to connect to the prior, prompting a search for nearby peers and establishing the routing table used by the DHT.
3. The DSR and target services are created and registered in the DHT, allowing for these to be resolved by other peers.
4. The test harness iterates through each available peer and uses the registry to execute a search for each target service, recording the number of iterations and the time for each search operation to complete.

Each DSR search operation begins by deriving a TID and issuing a DHT lookup for tertiary pages and then the primary page for each discovered service. Per the process described in Section 3, the number of DHT iterations (and, thus, network transactions) depends on the number of available peers, the distance between the target and requesting IDs, and the configuration of the DHT. As peer and service identifiers can be assumed to be randomly distributed, the superset of all peers and services is used to ensure the best- and worst-case performances are captured, and the execution time of each operation is measured and collected for analysis. For this evaluation, we fix the DHT configuration to k=16 and α=4 as values proportional to the scale of the networks under test, and we elide churn by allowing for the network to reach a steady state prior to executing each test.

**Figure 10:**
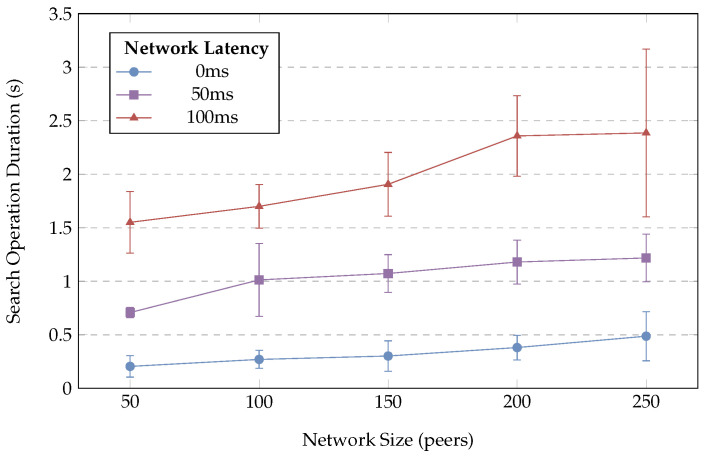
Search performance by network size and point-to-point latency.

**Figure 11:**
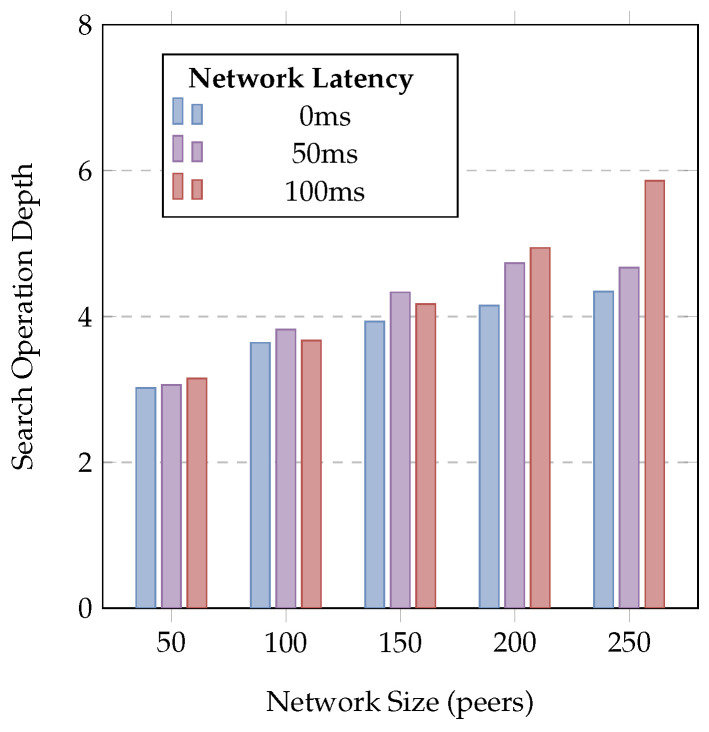
Average search depth by network size and point-to-point latency.

Where the performance of centralised approaches typically varies with the complexity of the query, the size of the database, and the number of coordinating registries, DSFs’ query performance depends primarily on the number of lookups required to perform a search (and the performance of these lookups). Figure 10 illustrates the average search operation duration for varying network sizes and latencies, with bars showing standard deviation to highlight variability between queries, while Figure 11 illustrates the average query depth required to resolve a query for each network size and latency. As the number of peers grows, the number of iterations required to locate peers near an ID increases, causing an increase in both the mean and variation of search durations, while increased network latencies cause an increase in both the duration and variation of search operations. This shows that across the tested network sizes, average query durations vary from 0.2 to 0.5 s with no injected network latency, 0.7 to 1.2 s with 50 ms of network latency, or 1.5 to 2.5 s with the worst-case 100 ms of network latency. It is expected that real-world performance will fall within these best- and worst-case bounds depending on network size and topologies. Table 1 provides a summary of related approaches to service discovery with published performance figures. This provides a baseline to contextualise DSRs performance against existing approaches; however, due to differences between testing methodologies and network models, these values are only suitable for broad comparisons. DSR provides similar query performance to existing works; however, as described in Section 3, DSRs’ trustworthy discovery mechanism requires 1+N DHT queries (where *N* is the number of matching services) to query the registry and then resolve information for each service, effectively doubling the execution time for a query with a single match when compared to untrusted approaches. In contrast to the lower-fidelity approaches to simulation typically used with decentralised technologies, this cluster-based evaluation demonstrates the correct operation of the distributed registry as well as the suitability of DSR for interactive use, achieving the performance requirements for real-time user interaction and allowing users to issue queries and discover IoT services using a decentralised infrastructure.

## 6. Evaluation

In contrast to existing centralised and decentralised registries, DSRs provide a trustworthy end-to-end approach to global service discovery and interaction. This approach dramatically simplifies the creation and sharing of service registries by decoupling registry instances from supporting infrastructure and allowing for multiple public and private registries to be deployed using the shared peer-to-peer network. Where centralised registries require the deployment of API and database servers alongside mechanisms for security and authentication, DSRs can be created using a one-line console command and then shared by providing users with a registry ID (and credentials for private instances), as demonstrated in Section 4. Decentralisation also allows for this supporting infrastructure to be constructed from heterogeneous edge devices in place of centralised cloud-based computing and storage. Overall, this decreases the cost and complexity of deploying and maintaining global service registries while addressing key challenges required to enable a global IoT. These key challenges are summarised in Table 2. DSRs also offer a valuable alternative to existing decentralised approaches to service discovery, addressing the need for trust, privacy, and access to data to enable future IoT applications. DSRs are compared to existing decentralised approaches in Table 3.

Where centralised registries require complex sharding or mirroring of services for *scalability* and *reliability*, decentralised approaches such as DSRs distribute storage and querying between peers in the decentralised network. This removes the single point of failure of centralised registries as well as allows relatively low-performance devices to create registries that can support high volumes of queries. Health-checking and duplication of registry data within the DHT ensure registry availability in the face of churn, while the distribution of data across available peers allows for a degraded operation in the case of limited connectivity or loss of substantial numbers of peers. However, due to the peer-to-peer nature of DSRs, internet connectivity is required to create and access registry instances, rendering these unsuitable for use in isolated networks. While DSF may, in the future, be extended to support isolated networks, this use case is orthogonal to the need for global discovery and adequately served by existing centralised registries.

DSRs’ adoption of content-based addressing using globally scoped and cryptographically verifiable service *identifiers* means that services can be consistently, uniquely, and verifiably addressed regardless of physical or network location. In contrast to existing registries, this provides a cryptographic association between registry entries and services to address the risk of service impersonation without requiring external certificate infrastructure. DSF-IoTs mechanisms for *specification* provide unambiguous descriptions of services and data allowing for these to be exchanged and interpreted, while peer-to-peer *communication* provides access to services and data across disparate physical networks and enables service mobility. Where existing registries must be paired with brokering and database infrastructures for accessing services and data, this provides an end-to-end approach that allows users to discover, understand, and interact with relevant services using the decentralised network. This decentralised approach also improves access to and availability of data by allowing for data queries and subscriptions for discovered services to be fulfilled by service replicas.

Where existing registries fail to address the need for *trust* and *privacy*, DSRs’ use of signature chains and cryptographic linking allows users to view registration history and evaluate registry trust by observing the historic operation of the registry. This addresses the temporal challenge of device discovery, allowing users to discover active devices using the DHT as well as to execute queries for a specific time period using the registry history. Object signing provides integrity protection and ensures that registry entries (TPs) can only be published by the registry service. Field-based encryption and one-way TID derivation support the creation of private registries on public peer-to-peer infrastructures as well as zero-trust registry operation whereby the registry has no access to query or registration information. Overall, this allows users to discover services while ensuring the authenticity of query results, service identities, and published data, providing end-to-end trust from the registry down to the data published by the service to allow services to be safely discovered and utilised for real-world applications.

DSRs allow users to search for relevant services using simple context-based queries; however, the adoption of a decentralised database does impose a performance cost. Where centralised registries provide a single omniscient point for query execution and support complex queries, decentralised approaches require iterative peer-to-peer operations to resolve queries to services, and DSRs only support exact queries. While the measurements in Section 5 demonstrate that the DSR’s query performance is suitable for interactive use, this decentralised approach imposes higher query latencies than are typical of centralised registries. We believe the benefits of decentralised registries discussed above outweigh this performance impact; however, it is proposed that future works investigate the optimisation of query operations to improve the performance of decentralised service discovery.

## 7. Discussion

DSRs provide a novel alternative to existing centralised and decentralised service registries. However, the adoption of peer-to-peer technologies introduces several complications. Registries depend on internet connectivity for registry operation, leaving these unsuitable for use on local networks as well as providing less-than-ideal best-case query performance. It is proposed that future works introduce support for the caching of local service information and registry replication to allow for isolated operations and offer improved query performance on local networks, as well as investigating performance improvements for the DHT to improve query operation and the integration of techniques to support range-based and multi-attribute queries via a DHT overlay.

The cluster-based approach to peer-to-peer network emulation used for performance and operational testing supports the evaluation of distributed systems with relatively large numbers of peers using available and affordable commodity hardware. In contrast to more scalable low-fidelity approaches to simulation, this enables the end-to-end operational verification of distributed applications and provides a basis for the experimentation and evaluation of DSRs. This approach may be further refined to address several limitations:

- The use of all peers in the network to perform measurements causes test durations to increase with the size of the network; while the scale of test networks is currently limited by available hardware, in order to support larger test networks, approaches to sub-sampling and query parallelisation would enable the timely evaluation of larger networks.
- The testbench currently elides churn and packet loss which can impact the registry performance and the bandwidth required to maintain registry entries. Future improvements may include network sampling to measure performance and maintenance bandwidth under varying network conditions as well as the introduction of mechanisms to mitigate churn in the DHT [46,47].
- As network size and communication latencies change, the optimal values of α and *k* will vary. It may be desirable to investigate the variation of the DHT configuration for different networks; however, the general optimisation of the DHTs is considered outside the scope of this research.

Future expansions of the testbench may include models for the dynamic joining and leaving of peers to simulate churn while introducing mechanisms to prioritise network locality when storing and querying from the DHT. Due to the use of the full peer application and the sharing of computational resources between peers executed on a single host in the cluster, the size of the simulation is limited by available resources, primarily due to available memory, with the existing eight-node cluster supporting up to 250 peers. This resource-sharing is a necessary compromise to provide a scalable approach to evaluating large networks with limited physical resources and may be mitigated by adding additional hosts to the cluster as well as prioritising high memory capacity when selecting hosts. Alternatively, higher-performance amd64-based hosts could be used; however, this significantly increases the cost of deploying such a testbed.

## 8. Conclusions

We present Decentralised Service Registries (DSRs), a decentralised approach to global IoT service discovery using a novel S\Kademlia-based DHT and overlay. DSR builds on DSF and DSF-IoT to provide an end-to-end alternative to the existing centralised, siloed, and vendor-controlled IoT infrastructure for sharing, searching for, and interacting with IoT services. Where existing centralised registries require the deployment and maintenance of server infrastructure, and decentralised approaches typically fail to address the need for multi-tenancy, trust, and privacy, DSRs support the creation and use of public and private registries using common peer-to-peer DSF infrastructures. This allows users and organisations to simply and safely create and share public or private registries to best meet their needs, removing the need for IoT device vendors or other organisations to deploy and maintain specific registry infrastructure while addressing key IoT challenges to meet the requirement for context-based global discovery in the IoT.

Coupled with DSFs, support for service interaction and DSF-IoTs’ unambiguous specification for IoT services, DSRs provide an end-to-end approach to global service discovery and interaction, allowing users to safely share, find, and interact with IoT services in a global context. This operation is demonstrated through the creation and use of a registry to share and discover real-world IoT devices in Section 4, highlighting the end-to-end operation from querying for services to accessing service information and data. A novel cluster-based testbench is developed to enable a high-fidelity emulation of the peer-to-peer system by allowing virtual networks of varying sizes to be operated and evaluated using readily available and low-cost hardware. Where low-fidelity modelling provides scalable predictions of system performance, this cluster-based approach enables high-fidelity testing and validation of the real-world operation and performance of decentralised systems. This testbench is used to qualify the operation and performance of DSR across a variety of network sizes with varying communication latencies, emulating the operation of real-world peers communicating via the internet and providing a performance baseline for comparison with existing registries. DSRs have been evaluated against existing centralised and decentralised approaches, highlighting the functional and performance trade-off of each approach and demonstrating the suitability of decentralised registries to address the need for global service discovery in the IoT.

We believe that decentralisation is key to enabling a future pervasive IoT, moving from centralised, siloed, and isolated infrastructure to reliable and privacy-preserving peer-to-peer architectures that empower individuals and organisations to safely create and share services to best meet their goals. DSRs are a step towards that future, enabling the global sharing and use of services to support future IoT applications that span physical and organisational siloes. Future works are expected to continue this evolution through the development of user-friendly interfaces for interacting with registries and services, and the deployment of wide-scale networks of DSF-IoT devices to collect and share data and provide a platform for new IoT applications. DSF (with support for DSRs) is published as open-source software to support further research and real-world applications.

## Figures and Tables

**Table 1:** Resource discovery models with query performance.

Approach	Performance	Context
Cirani et al. [35]	200–500 ms	Location-based DHT, 0–1000 nodes with network delays via Cooja.
Tanganelli et al. [40]	250–320 ms	Fog-based DHT, 20–100 nodes with a constant 80 ms network latency.
Jia et al. [30]	230–430 ms	Centralised resource matching, algorithm-dependent.
Gomes et al. [31]	250–500 ms	Federated, queries executed across 1–5 repositories.
Kamel et al. [38]	50–850 ms	Region-based DHT, 10,000 nodes with 2 ms (local)–120 ms (regional) network latency.
Zorgati et al. [39]	190–210 ms	Clustered SON + DHT, 50 nodes, network latency not modelled.
DSR	700–1200 ms	DHT discovery and fetch, 50–250 nodes with 50 ms nominal network latency.

**Table 2:** Centralised vs. decentralised service registries.

	Centralised	Decentralised (DSR)
Identification	IP or DNS-based, inconsistent and limited to network or vendor scopes. Requires TLS and supporting infrastructure for verification.	Content-based, globally scoped, and cryptographically verifiable, addressing enabling trust and mobility.
Specification	Must be paired with external mechanisms for service description and data encodings.	Includes unambiguous, self-contained, and verifiable descriptions of service and data.
Communication	Typically out-of-scope for registry implementations while existing approaches are limited to interaction within a network or vendor ecosystem.	Opportunistic local and global service interaction via peer-to-peer infrastructure while retaining end-to-end privacy and security properties.
Scalability	Limited by the performance of the registry server, may be improved by mirroring or sharding servers at the cost of operational complexity.	Storage and queries are distributed across communicating peers, allowing constrained peers to create registries that support high volumes of queries.
Reliability	Centralised registries may be deployed on isolated or non-internet-connected networks; however, they typically offer a single point of failure.	Resilient to communication and service interruptions; however, internet connectivity is required to interact with the peer-to-peer network to execute queries.
Trust	Must be paired with TLS for transport security, no object verification or CT-like mechanisms for establishing registry trust or associating registry entries with services and data.	Registry entries are signed and linked to ensure integrity and create a verifiable history for each registry while ensuring the authenticity of services and published data.
Privacy	Registries have full access to service information and require out-of-band mechanisms for access control.	Supports the creation of private registries as well as zero-knowledge registration and querying.

**Table 3:** A comparison of decentralised approaches to service discovery.

	Registry			Service		
Approach	Trust	Privacy	History	Identification	Specification	Communication
Paganelli et al. [33]	✗	✗	✗	✗	✗	✗
Cirani et al. [35]	✗	✗	✗	✗	✓ ^1^	✓ ^2^
Li et al. [34]	✗	✗	✗	✗	✗	✗
Tanganelli et al. [40]	✗	✗	✗	✗	✓ ^1^	✓ ^2^
Kamel et al. [37,38]	✗	✓	✗	✗	✗	✗
Zorgati et al. [39]	✗	✗	✗	✗	✗	✗
DSR	✓	✓	✓	✓	✓	✓

✓ meets requirements. ✗ does not meet requirements. ^1^ Partial CoRE-based specification only. ^2^ No access to historic data.

## Data Availability

The data presented in this research is available alongside the software and tooling used for simulation and evaluation at https://github.com/dist-svc/dsf and https://github.com/dist-svc/eval (accessed 22 Feburary 2024).
